# Low Vitamin D Levels in Patients with Irritable Bowel Syndrome of a Tertiary Care Hospital: A Descriptive Cross-sectional Study

**DOI:** 10.31729/jnma.7070

**Published:** 2021-10-31

**Authors:** Abashesh Bhandari, Ashlesha Chaudhary

**Affiliations:** 1 Department of Internal Medicine, Nepal Medical College and Teaching Hospital, Jorpati, Kathmandu, Nepal; 2 Nepal Medical College and Teaching Hospital, Jorpati, Kathmandu, Nepal

**Keywords:** *irritable bowel syndrome*, *prevalence*, *vitamin d deficiency*

## Abstract

**Introduction:**

Vitamin D deficiency is one of the common disorders prevalent among both developed and developing countries. Irritable Bowel Syndrome has been linked to many diseases and conditions, one of them being Vitamin D deficiency. To the best of our knowledge, no study of vitamin D deficiency status has been done yet in Nepalese setup. This study aims to find out the prevalence of low levels of Vitamin D in patients with Irritable Bowel Syndrome attending the outpatient department of a tertiary care hospital.

**Methods:**

This descriptive cross-sectional study was conducted in patients with Irritable Bowel Syndrome attending the outpatient department of a tertiary care hospital in Kathmandu, Nepal between November 2020 and July 2021. Ethical approval was taken from the Institutional Review Committee of Nepal Medical College and Teaching Hospital (Ref No: 027-077/078). Convenient sampling was done. The collected data was entered and analyzed in Microsoft Excel. Point estimate at 95% confidence interval was calculated along with frequency and proportion for binary data.

**Results:**

Out of a total of 71 patients with Irritable Bowel Syndrome, the prevalence of low levels of vitamin D was 44 (61.97%) (95% Confidence Interval= 50.67-73.26). Out of the patients with low vitamin levels, insufficiency was seen in 23 (52.27%) and deficiency was seen in 21 (47.72%).

**Conclusions:**

Our study found the prevalence of low Vitamin D levels among patients with Irritable Bowel Syndrome to be lower when compared to other studies.

## INTRODUCTION

Irritable bowel syndrome (IBS) is a common health problem with disorganized bowel function which affects the quality of life characterized by abdominal pain or discomfort along with altered bowel habits.^1^ Studies have shown various comorbidities likely somatic pain syndromes, other gastrointestinal problems, and other psychiatric illnesses to be associated with IBS.^2-5^

Recent studies have proposed a relationship between low vitamin D and IBS which could be evident by its role as an immune modulator, anti-inflammatory, and anti microbial agent.^6-8^ In addition, the vitamin D receptors (VDR) are expressed in the gut affecting it's function, motility, and IBS symptoms.^9^

This study aims to find out the prevalence of low vitamin D levels in irritable bowel syndrome among the patients presenting to the Department of Internal Medicine of a tertiary care hospital.

## METHODS

This is a descriptive cross-sectional study conducted among patients with IBS presenting to the Department of Internal Medicine of a tertiary care hospital between November 2020 and July 2021. Ethical approval was taken from the Institutional Review Committee of Nepal Medical College and Teaching Hospital (Ref No: 027077/078).

Patients who had given their consent, fulfilled the ROME IV criteria, and were of age above 15 years were included in the study and those with a history of recent use of antibiotics(in the last 4 weeks), gut surgery or radiation, coeliac disease, pregnancy or chronic illnesses were excluded. Convenience sampling was done.

The sample size was calculated by using the formula,

n = Z^2^ × p × q / e^2^

  = (1.96)^2^ × 0.82 × (1-0.82) / (0.09)^2^

  = 70.0024 ~ 71

where,

n = Sample size,
Z = 1.96 at 95% Confidence Interval,
p = Prevalence of Vitamin D Deficiency in Patients with Irritable Bowel Syndrome based on past study.^10^
q = 1 - p
e = margin of error, 9%

Detailed history and clinical examination were done. Patients were diagnosed with Irritable Bowel Syndrome (IBS) by using ROME IV criteria:

Recurrent abdominal pain on average at least 1 day/week in the last 3 months and fulfilling two or more of the following criteria:

1. Relation with defecation
2. Associated with an alteration in the frequency of stool
3. Associated with an alteration in the form (appearance) of stool

(Present for the last 3 months with symptom onset being at least 6 months before diagnosis)^10^

Informed consent from these patients was taken and serum Vitamin D levels were measured. Around 5ml of whole blood, which was free from hemolysis was initially centrifuged at 3000 rates per minute at 10minutes. The separated serum was analyzed for vitamin D levels using an automated analyzer. Low levels of Vitamin D were defined by using the following criteria as per the 25(OH) D concentration:

1. Vitamin D sufficiency : greater than 20ng/mL (50nmol/L)
2. Vitamin D insufficiency : 12 to 20ng/mL (30 to 50nmol/L)
3. Vitamin D deficiency : less than 12ng/mL (30nmol/L)

The data were entered into Microsoft Excel and descriptive statistics were calculated. Point estimate at 95% confidence interval was calculated along with frequency and proportion for binary data.

## RESULTS

A total of 71 patients with Irritable Bowel Syndrome were included in the study. Among them, low vitamin D levels were found in 44 (61.97%) (50.67-73.26 at 95% Confidence Interval. The mean of the low vitamin D levels among the patients with irritable bowel syndrome was found to be 11.81±4.18. The mean age of the patients with low vitamin D levels was found to be 36.4 years. Out of the patients with low vitamin levels, insufficiency was seen in 23 (52.27%) and deficiency was seen in 21 (47.72%) (Table 1).

**Table 1:** Distribution of patients according to gender (n = 44).

	Vitamin D Deficiency (n = 21) n (%)	Vitamin D Insufficiency (n = 23) n (%)	Total n (%)
Males	7 (33.33)	8 (34.78)	15 (34.09)
Females	14 (66.66)	15 (65.21)	29 (65.90)

These 44 (61.97%) patients who had low vitamin D levels were sub-classified based on their predominant stool pattern as IBS with constipation (IBS-C), IBS with diarrhea (IBS-D) and others (includes Mixed IBS and Unsubtyped IBS)^11^ as shown in the table below (Table 2).

**Table 2:** Subtypes of IBS (n = 44).

	IBS-D n (%)	IBS-C n (%)	Others n (%)
Vitamin D Deficiency (n = 21)	11 (52.38)	7 (33.33)	3 (14.28)
Vitamin D Insufficiency (n = 23)	13 (56.52)	7 (30.43)	3 (13.04)
Total (n = 44)	24 (54.54)	14 (31.81)	6 (13.63)

Out of these patients, the common clinical features were studied in the vitamin D deficient and vitamin D insufficient patients which included altered bowel habits, pain abdomen, lower back pain, weakness, and low mood (Table 3).

**Table 3:** Clinical features among patients of IBS with low Vitamin D levels (n = 44).

	Lower Back Pain n (%)	Weakness n (%)	Low Mood n (%)	Pain Abdomen n (%)	Altered Bowel Habits n (%)
Vitamin D Deficiency (n=21)	21 (100)	19 (90.47)	18 (85.71)	21 (100)	21 (100)
Vitamin D Insufficiency (n = 23)	23 (100)	20 (86.95)	17 (73.91)	23 (100)	23 (100)

Among the 44 patients with low levels of vitamin D in IBS, the study of the stool frequencies showed most common to be once in more than 2 days numbered as 18 (40.9%) (Table 4).

**Table 4:** Stool frequencies among patients of IBS with low Vitamin D levels (n = 44).

	0-2 times a day n (%)	3-4 times a day n (%)	More than 4 times a day n (%)	Once in more than 2 days n (%)
Vitamin D Deficiency (n = 21)	0 (0)	2 (9.52)	10 (47.61)	9 (42.85)
Vitamin D Insufficiency (n = 23)	4 (17.39)	9 (39.13)	1 (4.34)	9 (39.13)

## DISCUSSION

Ever since the association of vitamin D with various other systemic illnesses have been established, vitamin D has been the focus of discussion in the medical field. Even though the role of vitamin D deficiency in IBS has not yet been clearly evident, multiple studies are underway to clearly establish its role. A recent report has been remarkably successful in sparking a gush of interest from the scientific community and attention of the medical blogs as it showed a success in the treatment of IBS with large doses of oral vitamin D supplementation, further showing resolution of the associated anxiety and depression as well.^12^ However, to the best of our knowledge, this is the first study of low vitamin D levels among Irritable Bowel Syndrome in Nepal.

The present study found the prevalence of low Vitamin D levels among patients with Irritable Bowel Syndrome to be lower when compared to another study by Khayyat Y, et al.^7^ which detected vitamin D deficiency (here defined as <50nmol/L) among 82% of the patients with IBS. The mean serum level of 25(OH) D3 in IBS patients was 21±12nmol/L which was higher than our study's findings.

Similarly, another study demonstrated the insufficiency of Vitamin D in patients with IBS among 67.3%. This finding was comparable to our study which although they had neglected to define their threshold of vitamin D insufficiency.^13^ In a US-based study, medical records of 1000 IBS patients were studied which reported that 72% of women and 3% of men with IBS showed a serum concentration < 30nmol/L. The mean of vitamin D level was 25.05nmol/L which was significantly higher when compared to our study.^14^

In another study, majority of participants had baseline 25OHD levels that are considered insufficient/severely deficient with an overall sample mean 25OHD of 15.3±7.9ng/mL and 81.8% of IBS-C (n = 7), 70% of IBS-D (n = 9) and 81.6% of IBS-M (n = 24) with <20ng/mL circulating 25OHD levels. This prevalence was higher when compared to our study.^15^

The main limitation of this study was the small sample size which shows the need of larger studies. This study is conducted in a single center so it cannot be generalized and the findings of this study is limited to our population only. Also, being a descriptive Cross-sectional study, causality cannot be established and there is a need of further studies for the same. Some of the cases of IBS may have been missed which would affect the overall validity of the study.

## CONCLUSIONS

The prevalence of low levels of Vitamin D in patients with Irritable Bowel Syndrome included in our study was lower than other similar studies conducted in the past. Higher studies and well powered trials are required to be conducted further in order to associate low levels of Vitamin D with different gastrointestinal disorders. The need for this is emphasized as vitamin D deficiency is a preventable and treatable condition which if addressed correctly could lower the morbidity associated with it.
